# New trends in the treatment of open-angle glaucoma: a critical review

**DOI:** 10.1007/s10792-025-03773-2

**Published:** 2025-09-24

**Authors:** Aigean Bilal, Farah Constantin, Sergiu Chirila, Tony Hangan

**Affiliations:** 1 Emergency County Clinical Hospital “Sf. Apostol Andrei”, Constanta, Romania; 2 Ovidius University, University Alle No.1, 900470 Constanta, Romania

**Keywords:** Open-angle glaucoma, Laser therapy, MIGS, Surgical glaucoma treatment, Filtration surgery, Neuroprotection

## Abstract

**Purpose:**

This critical review explores current and emerging strategies for the management of open-angle glaucoma (OAG), highlighting pharmacological, laser-based, and surgical innovations. It aims to synthesize recent evidence, assess real-world applicability, and evaluate future directions in personalized glaucoma care.

**Methods:**

A comprehensive literature search was conducted across PubMed, Scopus, Web of Science, and Cochrane Library databases for English-language publications between January 2000 and May 2025. Studies on first-line therapies, combination treatments, microinvasive glaucoma surgery (MIGS), laser modalities, and novel drug delivery systems were included. The review presents an overview of significant advancements and future directions in glaucoma treatment.

**Results:**

Prostaglandin analogs remain the cornerstone of medical therapy, while fixed combinations and sustained-release implants improve adherence. Selective laser trabeculoplasty (SLT) has gained traction as a first-line treatment in mild OAG, though its effectiveness in advanced stages is limited. MIGS procedures are increasingly used as safer alternatives to traditional surgery, especially when combined with cataract extraction. Affordable goniotomy techniques and suprachoroidal approaches offer promising solutions in resource-constrained settings. Neuroprotection, vascular modulation, and caspase inhibition represent future adjunctive strategies. In Romania, glaucoma remains a major public health challenge, with limited treatment coverage and delayed diagnoses due to systemic gaps in education and screening.

**Conclusions:**

Management of OAG is shifting toward patient-centered interventions that balance efficacy, safety, and accessibility. Continued integration of novel therapies, surgical innovation, and real-world cost-effectiveness data is essential for optimizing treatment algorithms, particularly in under-resourced health systems.

## Introduction

Glaucoma is a term used to describe progressive and irreversible optic nerve damage, visual field loss, and eventually complete vision loss [1]. It is one of the leading causes of blindness worldwide among the active population in industrialized countries. It affects 2–3% of the population over 40, but up to 50% may be undiagnosed [2]. This disorder is primarily caused by elevated intraocular pressure, but there are other mechanisms involved that are incompletely elucidated at this time. Early diagnosis is essential for effectively managing and preventing associated comorbidities, and intraocular pressure currently remains the only modifiable risk factor. In this sense, research is focused on developing new noninvasive diagnostic methods and discovering new therapeutic targets to improve the prognosis and quality of life of those affected by this condition [3]. Moreover, the education and awareness of the population regarding glaucoma and the importance of regular screening are vital for reducing the incidence of blindness. In Romania, glaucoma is a significant public health issue, being the second leading cause of blindness in the country. Current estimates suggest that over 160,000 individuals are affected, yet fewer than 50% receive appropriate treatment. The lack of a centralized national registry, combined with limited access to screening and follow-up programs, hinders early diagnosis and long-term disease control.

Several factors contribute to this situation: the silent progression of the disease, insufficient public awareness of glaucoma symptoms and risks, poor adherence to therapy, and systemic gaps in preventive ophthalmologic care. Addressing these barriers through structured screening programs, improved health education, and consistent follow-up remains a critical priority for reducing preventable vision loss in Romania.

The American Academy of Ophthalmology recommends that all adults have at least one check-up until the age of 40, and for people over 40, annual check-ups are recommended. Specific risk groups require checks at shorter intervals, including increased intraocular pressure, advanced age, positive family history, African or Hispanic descent, small central corneal thickness, myopia, and genetic mutations [4]. Another anatomical factor contributing to glaucoma pathophysiology is age-related lens enlargement. As the lens increases in size, either physiologically with age or due to cataract formation, it can exert anterior pressure on the iris, reducing the depth of the anterior chamber and narrowing the iridocorneal angle. This mechanical crowding impairs aqueous humor drainage, leading to elevated intraocular pressure. Although most commonly associated with angle-closure glaucoma, this mechanism can also exacerbate open-angle glaucoma by promoting pigment dispersion, angle narrowing, or worsening outflow resistance [5]. Additionally, increased contact between the enlarged lens, iris, and zonular fibers can lead to pigment liberation from the posterior iris surface**.** The liberated pigment is then deposited into the trabecular meshwork, obstructing aqueous outflow and further raising intraocular pressure. This mechanism is particularly relevant in pigment dispersion syndrome and secondary open-angle glaucoma [6]. An essential but less-discussed factor is stress; there is a strong correlation between the progression of glaucomatous optic neuropathy and stress in patients with glaucoma [7].

The purpose of this article is to review the latest trends in the therapeutic management of open-angle glaucoma.

## Materials and methods

**Fig. 1:**
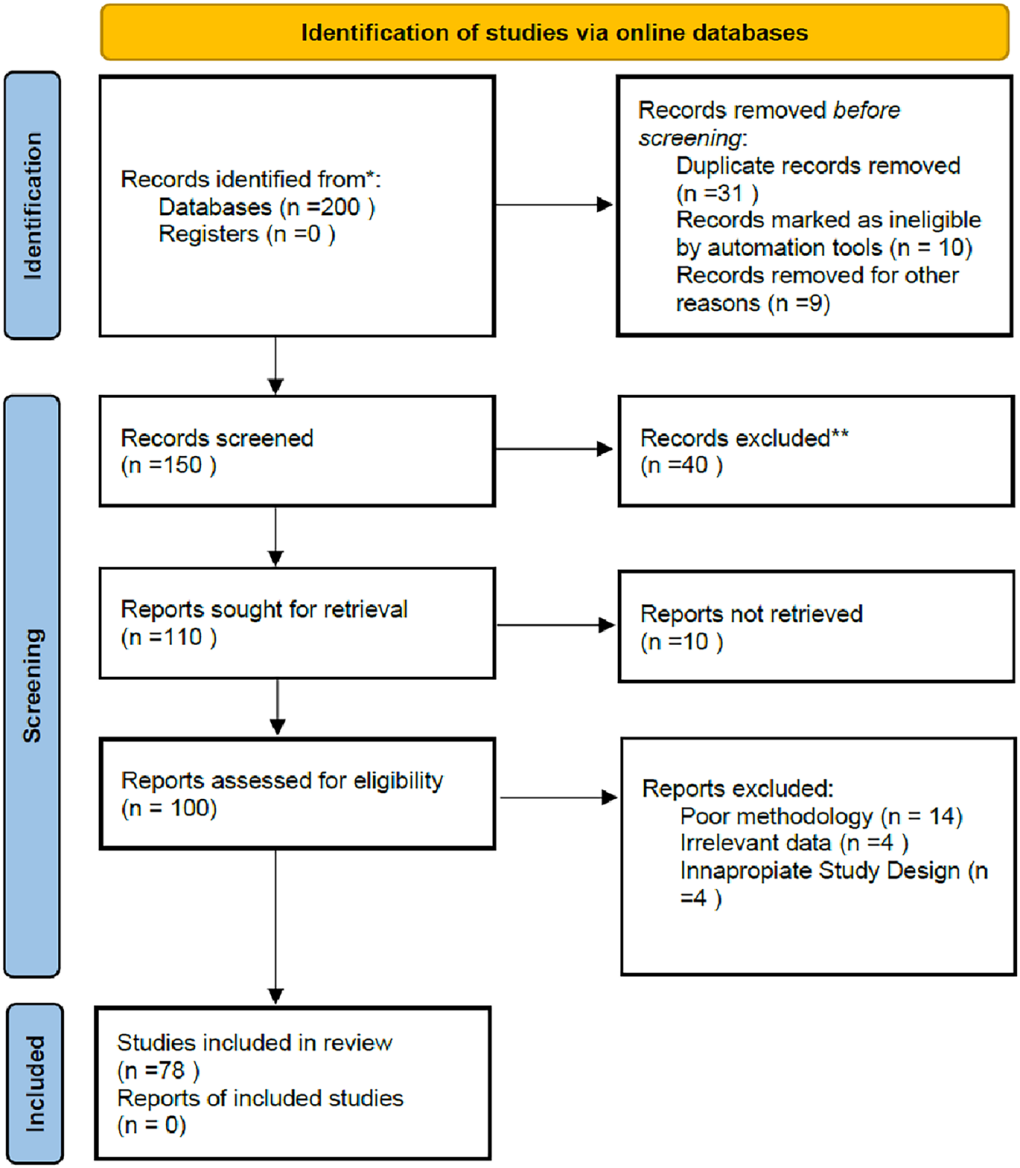
PRISMA flow diagram of the study selection process (* databases searched: PubMed, Scopus, Web of Science, Cochrane Library.** Exclusion criteria: non-English publications, conference abstracts, animal/laboratory research, duplicate datasets)

A systematic search of electronic databases, including PubMed, Scopus, Web of Science, Elsevier, and the Cochrane Library, was used to identify the studies underlying these new trends. Studies written in English between January 2000 and May 2025 were considered, using keywords such as open-angle glaucoma, glaucoma drainage implants, trabeculectomy, micro-invasive glaucoma surgery, and laser. Exclusion criteria were: non-English publications, conference abstracts, case reports, animal or laboratory research, and duplicated datasets. However, isolated case reports and preclinical studies were discussed narratively in the Future Perspectives section, but were not included in the systematic evidence synthesis. The findings highlight significant advances in glaucoma treatment, including the development of new drug classes, novel laser procedures, and innovative minimally invasive surgical techniques. This review presents an overview of the current state of glaucoma treatment and future directions (Fig. 1).

### Medical treatment of open-angle glaucoma

The only modifiable risk factor in slowing the progression of glaucomatous optic neuropathy is the decrease in intraocular pressure [8]. Medical therapy is usually the first-line approach recommended by international guidelines, as various combinations of topical hypotensive drugs can lead to satisfactory intraocular pressure targets [9]. If the target pressure is not reached, pressure can be further reduced using laser trabeculoplasty, a noninvasive technique to stop or to delay the surgical intervention [10].

The goal of treatment is to achieve a target intraocular pressure that prevents further glaucoma progression. For each patient, this target is established following the current stage of the disease, the potential adverse effects of the medication, and the current intraocular pressure [11]. In the early stages, monotherapy with a single drug class is typically recommended, such as prostaglandin analogs, beta-blockers, alpha-2adrenergic, and carbonic anhydrase inhibitors.

#### First-line therapies

- *Prostaglandin analogs* produce the most significant decrease in intraocular pressure [12] by decreasing filtration resistance and facilitating uveo-scleral drainage. Studies have shown that uveo-scleral drainage increases due to the relaxation of the ciliary muscle by dilating the spaces between the muscle fibers [13]. This effect is mediated by modulation of matrix metalloproteinase activity in ocular tissues [14]. According to a study conducted in Ontario, Canada, between 1994 and 2004, the use of drops reduced the need for trabeculectomy by half, closely connected with the introduction of prostaglandin analogs on the market [15].
- *Beta-blockers* decrease the synthesis of aqueous humor at the level of the ciliary body, thus reducing intraocular pressure. This therapeutic class was the most frequently used until the advent of prostaglandin analogs, which proved to be more effective with fewer adverse effects and a more substantial decrease in intraocular pressure. The best effects are obtained during the day, so it is recommended to use them in the morning [16]. Careful patient selection is recommended because there are several adverse effects, such as bronchospasm, hyperglycemia, bradycardia, or muscle weakness, so they must be replaced with another class of drugs. If using monotherapy does not reach the target eye pressure value, two classes of drugs can be used simultaneously, or we can add other classes, such as carbonic anhydrase inhibitors and alpha-2 agonists [17]
- *Alpha 2-agonists* increase the drainage of aqueous humor on the uveo-scleral pathway and decrease the production of aqueous humor and the pressure in the episcleral veins. Local adverse effects include eyelid retraction, conjunctival discoloration, xerostomia, fatigue and allergic reactions mainly due to benzalkonium chloride. Multiple studies suggest a neuroprotective effect on the optic nerve of brimonidine by preserving the visual field at four years compared to timolol, despite the same decrease in eye pressure[18, 19].
- *Carbonic anhydrase inhibitors* lower intraocular pressure by reducing aqueous humor production by approximately 50%. This class does not act via the uveoscleral outflow pathway. Local adverse effects include discomfort, stinging sensation, and burning.
- *Miotics* have been used for a long time in treating glaucoma, but their use has decreased dramatically with the advent of new, more powerful therapeutic agents with fewer side effects (Table 1).

**Table 1:** Medical treatment of open-angle glaucoma

Therapy Type	Examples	Mechanism	Approval Status
First-Line	Prostaglandins (latanoprost), Beta-blockers	↓ Aqueous production/↑ Uveoscleral outflow	Approved
Combination Therapies	Timolol + Dorzolamide, Latanoprost + Timolol	Mixed	Approved
Emerging Therapies	Netarsudil, Ripasudil, Trabodenoson, Intracameral bimatoprost implant	Trabecular outflow, neuroprotection, long-acting	Mixed

#### Combined therapeutic agents

Combining these drug classes results in a stronger hypotensive effect, but it also increases the risk of adverse effects. For this reason, the simultaneous use of several types of drugs leads to a decrease in patient compliance [20]. For these reasons, *fixed combinations* of them were created. Some examples available are dorzolamide plus timolol, latanoprost plus timolol, travoprost plus timolol, brinzolamide plus timolol.

To reduce systemic absorption through the nasal mucosa and nasolacrimal duct, patients are advised to close their eyes and gently press the inner canthus with a finger after instillation. However, following glaucoma surgery, punctal occlusion should be used with caution in the early postoperative period, as it may increase drug absorption and lead to ocular hypotony. This can help lower the risk of experiencing systemic side effects [21]. In the past, benzalkonium chloride was used to preserve the quality of the products, but this produced unwanted local effects affecting the ocular surface. For this reason, the so-called "preservative-free" products appeared on the market, leading to fewer complications such as dry eye syndrome.

The low patient compliance with the use of drops led to a new approach, namely the intracameral injection of slow-release medicine. In 2020, the FDA approved using the bimatoprost intracameral implant, which was as effective in lowering intraocular pressure as topical administration over 4–6 months [22]. The intracameral bimatoprost implant represents a significant advancement in glaucoma therapy by providing sustained intraocular pressure reduction through a biodegradable drug delivery system. This innovative approach enhances treatment adherence, minimizes the need for daily topical medications, and reduces the incidence of prostaglandin-related ocular surface side effects [23]. Ongoing drug development has led researchers to investigate new classes of intraocular pressure-lowering agents.

#### Emerging and investigational therapies

In order to enhance the structure and clarity of this review, the section on novel therapies has been organized according to the regulatory status and strength of supporting evidence for each treatment. Specifically, the therapies are grouped as approved (with regulatory authorization and Phase 3 clinical data), investigational (currently undergoing clinical trials), and speculative (based mainly on preclinical research or early exploratory studies). This classification helps to better understand the clinical relevance and current applicability of each option, while also highlighting promising directions for future research.

- *Rho kinase (ROCK) inhibitors* have emerged as a novel class of antiglaucoma agents that act primarily by enhancing aqueous humor outflow through the trabecular meshwork and Schlemm’s canal [24]. Netarsurdil, a dual noradrenaline and Rho kinase inhibitor, increases trabecular outflow by dilating juxtacanalicular tissue, dilating the episcleral veins, and relaxing the ciliary muscle [25]. Netarsurdil 0.02% ophthalmic solution is the first drug in this class approved in the USA by FDA, achieving IOP reduction comparable to prostaglandin analogs with once-daily dosing. The effectiveness of monotherapy was demonstrated in phase 3 studies that concluded with an average decrease in intraocular pressure by 6.1 mmHg during one year [26]. Beyond their IOP-lowering efficacy, ROCK inhibitors exhibit potential neuroprotective and antifibrotic effects, with implications for optic nerve head perfusion and postoperative scarring in glaucoma surgery [27].
  The combination of netarsurdil and latanoprost available in the US demonstrated an additional reduction in intraocular pressure of 2 mm Hg compared to latanoprost monotherapy. The most frequent complication reported was conjunctival hyperemia, generally mild and dose-dependent [28]. The drug's safety, tolerability, and efficacy make this molecule a promising new therapeutic option, either as monotherapy or in combination with other ocular hypotensive agents for treating primitive open-angle glaucoma and intraocular hypertension syndrome [29].
  Ripasudil 0.4%, a selective Rho kinase inhibitor, has demonstrated efficacy in lowering intraocular pressure when added to existing maximum tolerable medical therapy in patients with primary open-angle glaucoma inadequately controlled despite maximal tolerated medical therapy. In a prospective clinical study involving 42 eyes, the introduction of ripasudil resulted in an average IOP reduction of 16.8% over a 3-month period, with a statistically significant mean decrease of 2.9 mmHg (*p* < 0.001). Nearly 43% of participants reachend their target IOP following adjunctive treatment. The medication was generally well tolerated; the most frequently reported adverse event was conjunctival hyperemia (observed in over half of the patients), which appeared shortly after administration and typically resolved within hours. Mild blepharitis occurred in isolated cases [30].
- *Trabodenoson* is a potential new molecule effective in treating hypertension and open-angle glaucoma. Its mechanism is binding to the adenosine A1 receptor, which enhances intracellular signaling by activating metalloproteinases in the trabecular meshwork. These enzymes degrade glycosaminoglycan deposits that contribute to outflow resistance through the trabecular meshwork [31]. This drug is still in clinical trials and has not yet been approved.
- *Latanoprostene bunod* is a prostaglandin analog that has a dual mechanism of action. The first component is latanoprost, a prostaglandin analog that has been used successfully for over two decades as the first line of treatment in primitive open-angle glaucoma, and the second is butanediol mononitrate, which releases nitric oxide. It relaxes the cells in the trabecular network, widens the intercellular spaces, and increases the outflow. According to research, it is more effective than timolol, leading to a reduction in intraocular pressure of approximately 32% with just one daily administration, and compared to latanoprost alone, it resulted in an additional 1.2 mmHg reduction in IOP. This reduction is clinically relevant, as each 1 mmHg decrease in IOP is associated with an approximately 10% lower risk of visual field deterioration [32].

A significant increase in vessel density was observed across all retinal layers analyzed by OCT angiography. Notably, in the superficial retinal layer, vessel density in the superior and nasal quadrants was significantly lower in patients with pseudoexfoliative glaucoma compared to those with primary open-angle glaucoma after one month of treatment (P = 0.038 and P = 0.019, respectively) [33]. This molecule was approved in November 2017 by the FDA for the treatment of primitive open-angle glaucoma in the USA after the results presented in phase 3 trials [34].

### Future perspectives on new drugs

- Agents with novel mechanisms of action
  Lowering intraocular pressure has always been the primary way of treating glaucoma. However, despite adequate IOP control, glaucomatous optic neuropathy may continue to progress due to ischemic suffering and retinal ganglion cell apoptosis [35]. Thus, the decrease in intraocular pressure will not be sufficient for a certain category of patients, as multiple clinical trials are underway to evaluate the efficacy of these novel therapeutic candidates. Mitrogenol, Vitamin E, N-acetylcysteine, memantine, glutathione, rutin, forskolin, vitamins B1 and B2, marijuana, nimodipine, and erythropoietin were included in the studies. The excessive accumulation of glutathione leads to overstimulation of NMDA receptors, resulting in retinal ischemia [36]. Memantine leads to the selective blocking of this receptor, which could have a neuroprotective effect, but studies show mixed results. Another NMDA receptor blocker had a more substantial neuroprotective effect than memantine in studies on retinal ganglion cells.
  Mitrogenol is a dietary supplement with an effect on increasing blood supply to the optic disc and thus prevents ischemia. In the study led by Steigerwalt et al., a drop in intraocular pressure of 2.2 mm Hg was observed in the treated group over six months. The study concluded that a better blood supply to the optic disc can prevent glaucoma [37]. Vitamin E and other antioxidants have been included in various studies to combat oxidative stress at the cellular level. Vitamin E deficiency appears to be associated with premature retinal ganglion cell death in mouse models. The group of mice fed without the addition of vitamin E had significantly higher lipid peroxidation than those fed with the addition of vitamin E [38].
  Marijuana has been studied through its effect of increasing the drainage of the aqueous humor in the uveoscleral pathway. According to a case report published in 2005 that included a patient, marijuana was used as a first-line therapy due to poor tolerance and inadequate response to conventional treatments. Intraocular pressure decreased from 30 mm Hg to 15 mm Hg just by smoking the plant [39]. According to a randomized, double-masked study, sublingual and topical administration of cannabidiol did not decrease intraocular pressure. Therefore, marijuana cannot yet be recommended as a therapeutic option for glaucoma in the absence of more robust clinical evidence [40].
- Neuroprotection and vascular modulation
  Recalling again the involvement of the vascular factor in the etiopathogenesis of primitive open-angle glaucoma, the hypothesis of vascular contraction in the decrease of blood flow at the level of the optic papilla and the increase of intraocular tension was proposed. Vascular contraction can also cause hemorrhages. Nimodipine, a calcium channel blocker, has been shown to increase optic disc perfusion and enhance contrast sensitivity [41], and better visual field results [42], compared to placebo. The proposed new drugs have shown promising results even if they are in preclinical phases. Monotherapy is only sometimes the answer in treating glaucoma, but rather a more comprehensive approach by understanding the pathophysiology of the disease is needed.
  Beyond intraocular pressure reduction, neuroprotection has emerged as a key focus in glaucoma research. Caspase-mediated apoptosis plays a central role in retinal ganglion cell (RGC) loss in glaucoma, independent of intraocular pressure. Experimental models have shown that direct inhibition of caspase-2, -3, and -9 results in substantial RGC preservation, with up to 80% improvement in cell survival and delayed optic nerve degeneration. These findings support caspase inhibition as a promising adjunctive strategy in glaucoma therapy[43]. Future discoveries regarding neuroprotection must establish their role in glaucoma management.
- Sustained-release delivery systems
  Besides the intracameral bimatoprost implant already mentioned, the development of other sustained-release delivery systems represents a significant advancement in glaucoma therapeutics, addressing patient compliance challenges while maintaining consistent drug levels. In December 2023, the FDA approved the iDose TR implant, a first-of-its-kind biocompatible titanium intracameral sustained-release implant. The iDose TR ensures constant 24/7 medication release, with FDA approval based on two Phase 3 pivotal trials involving 1150 subjects. Both trials achieved primary efficacy endpoints, demonstrating IOP reductions of 6.6–8.4 mmHg versus 6.5–7.7 mmHg in controls through 3 months. Long-term data shows 81% of iDose TR patients were medication-free at 12 months, with implants lasting 4–5 years and safely exchangeable.
  These platforms include biodegradable implants, microparticle formulations, nanotechnology-based carriers, intravitreal systems, contact lens-based delivery, and punctal plugs, providing controlled drug release from weeks to months. Managing glaucoma with sustained-release procedural pharmaceuticals has transformational potential, reducing the burden of drop instillation for patients [44] (Table 2).

**Table 2:** Overview of emerging and investigational therapies for open-angle glaucoma

Therapy	Type/mechanism	Regulatory status	Evidence level
Netarsudil	Rho kinase inhibitor (↑ trabecular outflow)	FDA-approved (USA)	Phase 3 RCTs (ROCKET-1 & 2)
Latanoprostene bunod	NO-donating prostaglandin analog	FDA-approved	Phase 3 trials (VOYAGER, LUNAR)
Bimatoprost implant (Durysta)	Sustained-release prostaglandin	FDA-approved	Phase 3 studies (ARTEMIS 1 & 2)
Ripasudil	Rho kinase inhibitor	Approved in Japan only	Small prospective clinical studies
Trabodenoson	Adenosine A1 receptor agonist	Investigational	Phase 2–3 trials (ongoing)
Caspase inhibitors	Apoptosis inhibitors (neuroprotection)	Preclinical	Experimental animal models
Memantine	NMDA receptor blocker	Not approved for glaucoma	Mixed results in human studies
Mitrogenol	Improves optic nerve perfusion	Dietary supplement	Small human study with IOP reduction
Vitamin E, NAC, Glutathione	Antioxidants/reduce oxidative stress	Speculative	Preclinical and animal models
Marijuana/cannabinoids	Enhance uveoscleral outflow	Not approved	Case reports and pilot trials
Nimodipine	Calcium channel blocker—improves ocular blood flow	Off-label	Small randomized trials in NTG patients
iDose TR	Sustained-release travoprost implant (↑ uveoscleral outflow)	FDA-approved (2023)	Phase 3 trials

### Laser treatment

Medical therapy may be insufficient in some patients, who continue to exhibit glaucomatous progression despite treatment. SLT has increasingly been adopted as a first-line treatment for patients with mild open-angle glaucoma. However, its efficacy in moderate to advanced stages is more limited. In such cases, combined cataract surgery, MIGS procedures, or traditional filtration surgery may offer superior long-term outcomes [45]. The effectiveness of laser treatment in glaucoma decreases over time, and for patients who register the progression of the disease, surgical treatment can be an option [46].

In 1973, Krasnov described using a laser in treating the trabecular meshwork. He described the increase in aqueous humor filtration through the laser point made in the meshwork and, thus, the decrease in intraocular pressure [47]. Wise and Witter described in 1979 the effectiveness and safety of using the Argon laser in modeling the trabecular meshwork, called argon laser trabeculoplasty (ALT), on a group of patients with primitive open-angle glaucoma. The mechanism is not entirely understood, but it is believed that the laser causes minor local scarring, and thus, the intercellular spaces increase.

Another hypothesis is the biological one, through the release of cytokines that promote local phagocytosis and thus the increase in filtration. This technique was proposed as an alternative to filtering surgery. Sudden tensional increases and anterior peripheral synechiae have been described as complications of this technique. An improved technique called micropulsed laser trabeculoplasty has been shown to cause less damage to the trabecular meshwork than ALT, using less thermal energy [48].

Selective laser trabeculoplasty (SLT), first introduced in 1998, is currently the most widely accepted first-line therapy for open-angle glaucoma. This technique is less traumatic than ALT by selectively targeting the melanin pigment causing minimal collateral damage to surrounding tissues.

In the study led by Latina et al., 70% of the eyes on which SLT was performed showed a drop in intraocular pressure of at least 3 mm Hg after 26 weeks [49]. Subsequent studies confirmed decreases greater than or equal to 20% compared to baseline at 12 months [50]. In 2019, the LiGHT Trial concluded that the treatment of intraocular hypertension syndrome and primitive open-angle glaucoma through SLT is more cost-effective than the use of topical treatment, and 74.2% of patients did not require further treatment to control intraocular pressure during the period studied [51]. SLT has increasingly been adopted as a first-line option, rather than being reserved for cases unresponsive to maximal medical therapy. SLT offers several advantages: it eliminates the need for daily patient compliance, achieves IOP reduction comparable to drug therapy, is cost-effective, and reduces plastic waste from medication packaging [52]. Beyond its increasing role as a first-line treatment, SLT has also been critically evaluated against medical therapy in real-world practice. Comparative data indicate that SLT provides similar or superior intraocular pressure control compared to topical medications, with the added advantage of improving treatment adherence and reducing patient burden. While SLT eliminates the need for daily drug administration in many patients, especially those with mild glaucoma, some individuals with more advanced disease may still require adjunctive pharmacologic treatment. Furthermore, real-world studies emphasize that SLT ensures more consistent diurnal IOP control and is less affected by issues of compliance or ocular surface side effects commonly associated with chronic drop use. However, procedural access and initial costs may still pose limitations in certain healthcare systems.

Systematic reviews suggest that 360° SLT yields greater IOP reductions and higher success rates at 12-month follow-up compared to the 180° approach, without increasing complication rates to the 180° approach. Importantly, this broader treatment area does not appear to increase complication rates, reinforcing the 360° SLT protocol as a more effective and equally safe option for managing open-angle glaucoma and ocular hypertension [53].

Selective laser trabeculoplasty (SLT) remains an effective treatment option even after previous pressure-lowering interventions. In a retrospective study of 122 eyes, approximately 68% of patients did not require further surgical intervention at one-year post-SLT, and 58% maintained this stability at two years. Notably, baseline intraocular pressure was a significant predictor for retreatment (*p* = 0.005), while glaucoma type had no statistically significant influence, although patients with pseudoexfoliation glaucoma tended to require additional intervention earlier than those with primary open-angle glaucoma [54].

Direct Selective Laser Trabeculoplasty (DSLT) is a novel, non-contact, image-guided laser procedure designed to lower intraocular pressure by targeting the trabecular meshwork translimbally, without the need for a gonioscopy lens or coupling gel. Using an automated device, DSLT delivers 360° laser treatment through the sclera to the underlying trabecular meshwork, offering a standardized, operator-independent procedure that can be delivered by a wider range of trained personnel. Its design improves accessibility and ease of use compared to conventional SLT. Although the GLAUrious trial failed to demonstrate statistical non-inferiority of DSLT compared to conventional SLT at 6 months, DSLT demonstrated clinically meaningful IOP reductions sustained over 12 months, with a comparable safety profile. As an automated, non-contact, image-guided procedure, DSLT offers the potential to expand access to effective glaucoma treatment, particularly in settings where conventional SLT is limited by the need for specialized equipment and personnel [55].

Other laser technologies under investigation are titanium-sapphire laser trabeculoplasty and pattern-scanning trabeculoplasty, which uses infrared energy. It penetrates deeper into the trabecular meshwork up to the level of the internal wall of Schlemm's canal [56]. Early studies have shown a decrease in intraocular pressure by both methods, but large-scale studies are needed to demonstrate their effectiveness and safety [57].

Cyclophotocoagulation involves the use of a laser diode to selectively ablate ciliary body tissue. Two techniques were described: transscleral laser cyclophotocoagulation and endoscopic laser cyclophotocoagulation. The first technique is used for refractory glaucoma. Still, the second one can be used at the same time as the cataract surgery as the ciliary processes are better visualized in aphakic or pseudophakic eyes [58]. A long-term study demonstrated that the average drop in IOP in patients who underwent phacoemulsification surgery and endoscopic cyclophotocoagulation decreased by 3.5 mm Hg. In contrast, in the control group, where only phacoemulsification was performed, the average drop was 0.7 mmHg [59]. The micropulse diode is a new technology for aiming laser energy towards the ciliary body in short-term impacts. Published data on this technique remain limited, and further studies are necessary to establish its efficacy [60].

### Surgical treatment

Historical data indicate that the number of trabeculectomies decreased by approximately 53% in the USA between 1995 and 2004. Similarly, a 47% decrease was reported in Canada between 1995 and 2010. These reductions are largely attributed to the increased use of cataract surgery with IOP-lowering effects and the emergence of minimally invasive glaucoma surgery (MIGS), rather than solely to improvements in pharmacotherapy [61]. Despite the efficacy of trabeculectomy, the procedure is associated with various early and late complications, including ocular hypotony, shallow anterior chamber, hyphema, bleb-related infections, and endophthalmitis. These risks, along with the complex postoperative care required, have contributed to the shift toward safer**,** less invasive surgical options, particularly for patients in earlier stages of disease. MIGS has gained popularity by offering effective IOP reduction with fewer complications, faster recovery, and the possibility of being combined with cataract surgery.

#### Drainage implants

The technique involves inserting a silicone tube into the anterior chamber, diverting aqueous humor into the sub-Tenon’s space for systemic reabsorption into the systemic circulation. Initially, they were used in the past for the treatment of refractory glaucoma, but according to the American Glaucoma Society, the use rate of drainage devices increased by 184% between 1995 and 2004 [62]. The Ahmed–Baerveldt Comparison Study evaluated the safety and efficacy of using the Ahmed FP7 valve and the Baerveldt Implant. The study concluded that five years after the intervention, the intraocular pressure dropped from 31 to 32 mm Hg to 14.7 mmHg for patients who received the Ahmed valve and for the group with the Baerveldt implant to 12.7 mm Hg. The Baeveldt implant group required fewer adjuvant treatments during this period The Ahmed ClearPath valve was approved by the FDA and introduced in 2019 to manage glaucoma. The treatment of juvenile glaucoma reduced the mean intraocular pressure from 36 mm Hg preoperatively to 12.4 mm Hg 12 months after implantation[62]. In adult glaucoma, the multicenter study by Grover et al. demonstrated a reduction in mean intraocular pressure from 26.3 mm Hg preoperatively to 13.7 mm Hg 6 months after implantation. The most frequent complications were anterior chamber inflammation, ocular hypotony, cystoid macular oedema, and hyphema [63].

#### Microinvasive glaucoma surgery (MIGS)

Minimally invasive glaucoma surgeries (MIGS) are a new class of procedures suitable for patients eligible for stent implantation or traditional surgery. Having a better safety profile, these techniques can be used as the first intention before classical procedures. Many of these procedures are performed simultaneously with cataract surgery, with the patient already being subject to an operative risk. The patients low compliance with drug treatment, the intraocular pressure variation during the day, as well as more stable intraocular pressure control, make these techniques increasingly preferred. Minimally invasive procedures can be used in mild and moderate cases of glaucoma, in patients who do not tolerate topical treatment, in patients with primitive open-angle glaucoma, pigmentary as well as glaucoma associated with pseudoexfoliation syndrome, and after laser treatments. They favor the passage of aqueous humor through 3 alternative ways: Schlemm's canal (a more expansive drainage space is created), the subconjunctival space, and the suprachoroidal space. These procedures share common features: an ab interno approach (typically at the nasal trabecular meshwork), rapid postoperative recovery, favorable safety profiles, and moderate IOP-lowering efficacy [64].

Trabecular micro bypass stents have the advantage of creating a better flow of aqueous humor between the anterior chamber and the collecting ducts. These devices function by creating a microtrabeculotomy and maintaining patency of Schlemm’s canal. The iStent G1 stent, made of titanium and coated in heparin, is inserted gonioscopically after phacoemulsification of the opacified lens and administration of intracameral carbachol. It is inserted through direct penetration of the canal. According to a randomized study, patients who benefited from phacoemulsification and stent implantation had postoperative intraocular pressures 17% lower than the group that only underwent phacoemulsification, which showed only a 9% reduction [65]. Also, implantation of a single stent can reduce intraocular pressure by approximately 25% and multiple stents even by 44% in one year.

The second-generation iStent G2 is smaller and offers improved placement within Schlemm’s canal. In a study conducted by Fea et al., it was concluded that the stent is as effective as medication in controlling intraocular pressure, with a very good safety profile [66]. iStent Supra is a third-generation variant built to create a drainage path between the aqueous humor and the suprachoroidal space through a minimally invasive approach [67].

Hydrus microstent is produced from a nickel-titanium alloy, which forms a flexible stent. It dilates the Schlemm canal four times more than the physiological diameter [68]. Pfeiffer et al. compared the effectiveness of the implant at the same time as cataract surgery versus patients with open-angle glaucoma treated only with phacoemulsification. The result was a significantly more significant drop in intraocular pressure in patients implanted with a stent [69].

Ab interno trabeculectomy is a surgical technique that uses a microelectrocautery to ablate the trabecular meshwork. Studies have reported an acceptable level of safety. This technique has been demonstrated to lower intraocular pressure by approximately 40%, with indications also in angle-closure glaucoma. The failure of internal trabeculectomy does not negatively affect a future conventional intervention, so classic trabeculectomy can be performed if necessary [70]

The suprachoroidal Cypass stent was initially approved by the FDA in 2016. It creates a filtering path between the anterior chamber and the suprachoroidal space, traversing the sclera, but due to high losses of endothelial cells, it was withdrawn from the market in 2018 [71].

Following the withdrawal of the Cypass device from the market, innovation in suprachoroidal glaucoma surgery continues with two noteworthy approaches. The first, AlloFlo, employs a bio-interventional design involving a controlled cyclodialysis cleft reinforced with a biocompatible scleral allograft. Preliminary reports suggest a sustained reduction in intraocular pressure of up to 40% when combined with cataract surgery, along with minimal endothelial cell loss and favorable safety outcomes [72]. Although still investigational and limited to select US centers, AlloFlo exemplifies a synthetic-free method with potential for improved tissue integration.

The second approach is the intrascleral ciliary sulcus suprachoroidal microtube technique. This cost-effective strategy uses a flexible microtube inserted via the ciliary sulcus into the suprachoroidal space through a small intrascleral tunnel. Early cohort studies have demonstrated significant and sustained IOP reduction and decreased medication dependence in pseudophakic patients, with a safety profile comparable to existing MIGS devices [73]. Its affordability, ability to be performed standalone or combined with cataract surgery, and preliminary positive outcomes suggest a promising role in expanding access to suprachoroidal outflow surgery.

Subconjunctival filtering sutures allow aqueous humor to drain into the subconjunctival space, similar to classic trabeculectomy. The XEN stent is a smaller hydrophilic tube than traditional devices, giving it a lower chance of damaging the corneal endothelium. According to a prospective study, intraocular pressure decreased from 20.8 mm Hg to 14.4 mm Hg after six months to 13.1 mm after one year postprocedural [74]. To minimize the risk of fibrosis associated with bleb formation, mitomycin C is often applied intraoperatively.

In addition to commercially available devices for subconjunctival and trabecular bypass, affordable manual goniotomy techniques are being adopted in resource-limited settings to provide intraocular pressure control comparable to proprietary MIGS options.

Bent Ab-Interno Needle Goniectomy (BANG) is a low-cost alternative that uses a simple, bent 25-gauge needle to incise the trabecular meshwork. When combined with cataract surgery, BANG has been shown to reduce IOP by approximately 26% at 6 months, with safety and efficacy outcomes comparable to the Kahook Dual Blade (KDB) procedure, but at a fraction of the cost [75].

Similarly, Sinskey Hook Goniotomy utilizes a reusable surgical hook already available in most operating rooms. Studies report that this technique, when performed with cataract extraction, results in a ~ 20% reduction in IOP and a 71% decrease in medication use at 6 months postoperatively. Its reusability and accessibility make it particularly well-suited for healthcare systems where cost is a major barrier to MIGS adoption [76].

Recent studies have shown that phacogoniotomy combined with phacoemulsification provides significant intraocular pressure reduction and a decrease in medication burden in both medically controlled and uncontrolled primary open-angle glaucoma. These findings suggest that this procedure may be considered earlier in the therapeutic sequence rather than reserved only for refractory cases. Furthermore, the extent of goniotomy has emerged as an important variable: partial versus complete procedures demonstrated different efficacy and safety profiles, with broader incisions achieving greater pressure reduction but carrying a slightly higher risk of complications. This variability underscores the need to individualize the surgical approach, limited goniotomy may be sufficient in primary open-angle glaucoma, whereas more extensive or circumferential techniques may offer advantages in juvenile and secondary forms. Collectively, these data emphasize the ongoing debate regarding how early minimally invasive glaucoma surgery should be integrated into management and what degree of goniotomy is most appropriate for different patient subgroups[77, 78].

These techniques demonstrate that cost-effective innovation in MIGS is feasible and can expand surgical options for glaucoma management in underserved populations.

#### Nonpenetrating glaucoma surgery

It refers to procedures by which the anterior chamber is not opened, and the trabecular meshwork is preserved. Deep sclerotomy and viscocanalostomy are procedures popularized in the 1990 as a safer alternative to conventional trabeculectomy, associated with fewer complications [79]. Most studies agree that it has fewer adverse effects, but the long-term effectiveness remains uncertain. Their main advantage lies in a better safety profile, as they avoid full-thickness penetration and minimize the risk of postoperative hypotony. A major limitation of these techniques is the large learning curve and the difficulty of the technique [80].

#### Future directions

MIGS devices can be a good alternative for patients with mild or moderate glaucoma as they avoid the formation of a filtration bleb and its complications. However, the long-term success of these interventions depends on the absence of fibrosis around them through antifibrotic agents and biocompatible materials that do not cause a local tissue reaction [81]. Postoperative ocular hypotony occurs even after valve implants, so new devices are being developed to address this complication, such as passive and active valves [82]. The advantage of passive valves is their simple use and easier manufacturing compared to active ones, and active ones have the advantage of adjusting the resistance to filter the aqueous humor to achieve the desired intraocular pressure. An example of such a valve is the eyeWatch Implant, the first implant to adjust intraocular pressure. This valve functions similarly to a faucet, with IOP adjustment performed using the eyeWatchPen control device, which adjusts the filtration resistance [83]. According to a study that compared the safety and effectiveness of this device versus the Ahmed valve, no case of ocular hypotony was reported compared to the group that received the Ahmed valve, where the percentage was 33% [84]. However, as a novel device, its long-term safety and efficacy remain to be established by future clinical research [85].

## Conclusions

Open-angle glaucoma remains a leading cause of irreversible blindness worldwide, with delayed diagnosis and limited access to care continuing to undermine public health efforts. Recent advancements in both pharmacologic and procedural therapies have shifted the management paradigm toward minimally invasive, personalized, and adherence-friendly solutions.

Prostaglandin analogs, fixed combinations, and intracameral sustained-release implants have strengthened the pharmacological arsenal. SLT is now widely accepted as a first-line option in mild cases. Meanwhile, MIGS techniques, especially when combined with cataract surgery, offer effective IOP reduction with fewer complications and faster recovery. Innovative options such as BANG and Sinskey hook goniotomy provide viable low-cost alternatives in underserved regions. Suprachoroidal procedures like AlloFlo and Dr. Laroche’s microtube technique extend the boundaries of safe, accessible outflow enhancement.

However, many therapies remain investigational or lack long-term comparative data. Future research should aim to refine the therapeutic sequencing of these options, integrate neuroprotective and vascular-targeted strategies, and adapt treatment pathways to local healthcare realities. Optimizing glaucoma management will ultimately depend on the alignment of clinical efficacy, safety, affordability, and system-wide implementation.

## Data Availability

No datasets were generated or analysed during the current study.
